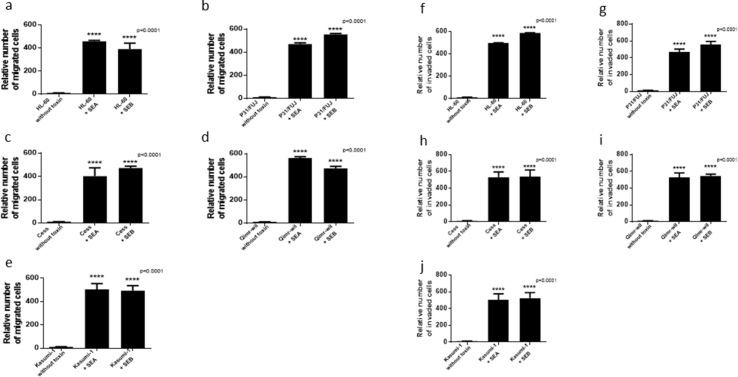# Enterotoxins A and B produced by *Staphylococcus aureus* increase cell proliferation, invasion and cytarabine resistance in acute myeloid leukemia cell lines

**DOI:** 10.1016/j.heliyon.2023.e19743

**Published:** 2023-09-02

**Authors:** Seyhan Turk, Hatice Yanpar, Ayriana Safari Baesmat, Secil Demirkol Canli, Olgu Erkin Cinar, Umit Yavuz Malkan, Can Turk, Ibrahim Celalettin Haznedaroglu, Gulberk Ucar

**Affiliations:** a Department of Biochemistry, Faculty of Pharmacy, Hacettepe University, Ankara, Turkey; b DS Nano and Biotechnology Product Tracing and Tracking Co., Ankara, Turkey; c Department of Medical Microbiology, Faculty of Medicine, Lokman Hekim University, Ankara, Turkey; d Molecular Pathology Application and Research Center, Hacettepe University, Ankara, Turkey; e Tumor Pathology, Cancer Institute, Hacettepe University, Ankara, Turkey; f Department of Hematology, Faculty of Medicine, Hacettepe University, Ankara, Turkey

**Keywords:** Acute myeloid leukemia, Staphylococcal enterotoxin A, Staphylococcal enterotoxin B, Cytarabine resistance, Invasion, Migration

## Abstract

As in the case of cancer, the risk of infection increases when the host's immune system is not working properly. It has been shown that toxins produced by the bacteria responsible for bacterial infections can alter the properties of cancer cells as well as their sensitivity to chemotherapy agents. *Staphylococcus aureus* (*S. aureus*) is one of the most prevalent pathogens in acute myeloid leukemia (AML) patients and it produces several virulence factors, including Staphylococcal enterotoxin A (SEA) and Staphylococcal enterotoxin B (SEB). Cytotoxicity, transwell migration, invasion assays, and various transcriptomic and gene set enrichment (GSE) analyses were used to determine how SEA and SEB alter cell proliferation, migration, invasion, and Cytarabine (Cyt) resistance in AML cell lines. The treatment of AML cell lines with SEA/SEB caused an increase in cell proliferation and Cyt resistance. Toxins enhanced the proclivity of cells to migrate and invade, with around 50% of cells in the presence of SEA and SEB. Transcriptomic and gene set enrichment analyses, and subsequent PCR validations showed dysregulation of immune related genes and genesets. Apparently, this allows AML cells to escape and survive the undesirable environment created by toxins, possibly via the ER stress signaling pathway. Therefore, SEA and SEB can significantly alter the characteristics of AML cancer cells and evaluation of alterations in responsible immune genes and pathways may be crucial for controlling the progression of cancer. In addition, our results suggest that there may be a strong interaction between the immune related pathways and the ER signaling pathway.

## 1 Introduction

Treatment related infections are significant contributors to morbidity and mortality in patients with acute myeloid leukemia (AML) [1]. The ever changing severe immuno suppression of AML patients, the extensive use of anti-infectives, and the complicated epidemiology of often resistant bacteria are revealing the importance and complexity of these infections [2,3]. Five to one percent of patients with acute leukemia and neutropenia develop a bloodstream infection due to risk factors including immunosuppression, corticosteroid use, and catheter insertion [4,5]. Such infections are usually caused by methicillin-resistant *Staphylococcus aureus* (*S. aureus*) (MRSA) and vancomycin-resistant enterococci (VRE) [2]. *S. aureus* is the main human pathogen responsible for a wide variety of infections, including skin and soft tissue infections, bacteremia, pneumonia, and various toxin-mediated diseases [6].

*S. aureus* infections have critical clinical effects on AML patients and an important cause of bacteremia in patients with hematological malignancies [7,8]. It is the most identified bacterial species in patients with acute leukemia and neutropenia, are fatal and associated with significant morbidity [9,10]. *S. aureus* include toxic shock syndrome toxin 1 (TSST-1), enterotoxin serotypes A to E (SEA, SEB, SEC, SED, SEE, and SEI) and enterotoxin-like serotypes G (SEIG), H (SEIH) and J to U (SEIJ-SEIU) [11]. As a result of the comparison of the sequences of the staphylococcal toxins, it was seen that they were divided into two groups. These are SEA, E and D group and SEB, Cl and C3 [12].

SEA and SEB have been well characterized in many immunological and neurobehavioral studies. They significantly increase the concentrations of IL-2, TNF-α and IFN-γ 2–4 h after intraperitoneal infection [13]. The effects of SEA are mediated by binding to the R-helix regions of MHC class Ⅱ molecules outside the peptide binding domain on antigen presenting cells (APC) and the variable region on T cell receptors. Then the toxin bridges the T cells and APCs. This event initiates the proliferation of a large number (∼20%) of T cells and eventually cytokine release occurs. High concentrations of cytokines contribute to the development of certain diseases [14]. SEB contains a large group of proteins produced by various types of bacteria, including staphylococcus, streptococcus, and mycoplasma. SEB is responsible for a number of pathophysiological changes in humans and mammals and triggers an extreme cellular immune response that leads to toxic shock. SEB, along with ricin and epsilon toxins, have been classified as Category B Priority Pantogenes by the National Institute of Allergy and Infectious Diseases [15].

Most cancers may affect, impair, or alter the functions of the blood and immune systems. For example, lymphoma and various types of leukemia originate from and eventually replace immune cells. These cells impair their response by blocking the regular functioning of our immune system [16,17]. It is the cancer treatment and microenvironmental changes, not the cancer itself, that alters the immune system. Cancer treatments can have both short-term and long-term effects. Long-term damage occurs when organs of the immune system, such as the spleen, are removed [18,19]. On the other hand, chemotherapeutic, immunotherapeutic, and radiation therapy cause short and medium-term immune system damage. Bone marrow and stem cell transplantation are critical treatment modalities, especially for patients with blood cancer. However, these treatments can cause the death of immune cells and increase the risk of infection. Infection that occurs during the therapy procedure can persist for months after the end of therapy [20]. Depending on the type and agent of the infection that develops during chemotherapy, the response to the drug used and the distinctive features of the disease may change. Due to the changes, the risk of infection increases in the defense mechanism of cancer patients. Cancer and cancer treatments can harm the defense system in many ways. Bacteria can cause genetic and characteristic changes in cancer cells, mostly with their external structure (e.g., capsule, cell wall, etc.) and the products they produce and release to the external environment (e.g., exotoxins) [2,21].

Cancer and its follow-up treatment, especially chemotherapy, may cause complications such as immunosuppression in patients, disruption of mucosal barriers as a result of long-term placement of vascular catheters [9,22]. Such complications also cause gram-positive bacterial infections, especially in cancer patients [2]. The evolution of cancer therapy and the changing epidemiology of major gram-positive pathogens highlight the need for further development in studies to understand the effect of bacteria that cause various infections in patients with cancer [19]. This means that current efforts are necessary to understand their relationship to cancer and their impact on the response to chemotherapy.

It is seen that bacterial exotoxins have a high potential to be effective on cancer cells through their strong effects on the immune system [16,17]. Within the framework of all these interests, this study aims to determine how two important enterotoxins of *S. aureus*, SEA and SEB, change cell proliferation, migration and invasion characteristics and chemotherapy resistance profiles of AML cancer cell lines, and to identify genes and pathways that may be associated or responsible for these changes.

## 2 Materials and methods

### 2.1 In vitro analyses

#### 2.1.1 Cell lines and cell culture

Human acute myeloblastic leukemia cell lines GDM-1, CESS, HL-60, and QIMR-WIL were purchased from ATCC (USA), Kasumi-1, P31/FUJ were purchased from Japanese Collection of Research Bioresources Cell Bank (Japan). Manufacturers authenticated the cell lines. P31/FUJ, GDM-1, HL-60, and CESS were cultured in RPMI-1640 (Sigma, (USA)) supplemented with FBS (% 10), P/S (% 1), and 200 mM L-glu (% 1). Kasumi-1 was cultured in RPMI-1640 supplemented with FBS (% 20). QIMR-WIL was cultured in DMEM (Sigma, (USA)) supplemented with FBS (% 10), P/S (% 1), and 200 mM L-glu (% 1). All cell lines were incubated at 5% CO_2_ and 37 °C in a humidified incubator.

#### 2.1.2 Cell proliferation assays

Growth percentages of each AML cell line was determined after treatment for 72 h with seven different (100, 50, 10, 5, 1, 0,1, 0.01 ng) Staphylococcal enterotoxins A (SEA) and Staphylococcal enterotoxins B (SEB) toxin concentrations compared to nonthreated ones (control group), by using ATP luminescence assay. Briefly, cells were treated with Cell titer-glo reagent and then the luminescence signal was recorded with a microplate luminometer (Turner Designs, CA, USA).

#### 2.1.3 Cell culture well migration image analysis

Cell lines were cultured in six well plates for one day. Next day cells were treated with SEA and SEB. After 72 h cells were monitored with an inverted microscope (Nikon Eclipse TS100). The length of the segmented cells edge was determined as the summed distance between all boundary points. Empty part of the image was selected by the Refine edge tool. It allowed us to precisely select empty areas of the well. The difference between the determined cells at t = 0 h and t = 72 h was reported as the cell displacement.

#### 2.1.4 Transwell migration and invasion assays

To evaluate migratory and invasive properties of AML cells, cells treated with SEA and SEB toxins. The only factor triggering migration and invasion in the wells is the presence of SEA and SEB. Migration and invasion tests were performed in Transwell plates (Lot21280416, Greiner bio-one, Germany) as previously described [23]. Cells were seeded in a six well plate. Unlike other techniques, such as scratch assays or removal of a physical barrier to accelerate migration seeding these cells does not involve the potentially complex factors of a physical stimulus that causes the cell to migrate or disrupt the migration surface. Cells (2.5 × 10^5^ cells/cm^2^) in 1000 μL DMEM supplemented with FBS (% 10), P/S (% 1), and 200 mM L-glu (% 1), were seeded onto 8.0 μm pore size, 425.4 mm^2^ surface area highly transparent polystyrene Transwell cell culture insert filter and incubated for 10 min at 37 °C and 5% CO_2_. After 10 min. 250 μl 10 ng SEA and 10 ng SEB were added to the cells in Transwell filters, separately. 3000 μL DMEM supplemented with 10% FBS, 1% P/S, and 1% 200 mM L-glu was added to the well which the filter would be placed in it and incubated for 4 h. A cotton applicator was used to carefully remove the medium and non-migrated cells above the membrane. 1 mL 3.7% formaldehyde was added to each well and the transwell filter was placed into fixation and incubated for 10 min. Transwell filter was washed two times with PBS. 100% methanol was added and washed again with PBS two times. Remaining methanol was removed. The transwell filter was allowed to dry and 500 μL of 0.2% giemsa was added to each well for staining cells that tended to migrate. After incubation for 10 min at room temperature, the giemsa was gently removed. Transwell filter were put in distilled water very carefully not to lose the fixed cells during washing and to remove excess giemsa. Then, transwell membrane was allowed to dry. Membrane was cut appropriately and placed on the hemocytometer. The cells were counted with an inverted microscope. Transwell migration test was modified to perform the invasion test. For invasion test, fibronectin (FB) from the extracellular matrix was added on top of the transwell membrane. FB was purchased from Heidelberg, Germany (1 mg/mL SERVA-21370). It was resuspended in 1 mL of PBS before use. The main stock concentration is 1 mg/mL. All wells were coated with FB the day before and incubated at 37 °C for 24 h. One day later, FB was removed, and the wells were washed three times with medium. Cells were seeded on the FB-coated inserts and the steps were repeated for the invasion test.

#### 2.1.5 Cytotoxicity assays

SEA and SEB, and Cytarabine (Cyt) were purchased from Sigma (USA). Cell titer-glo reagent was used for cell cytotoxicity assay (Promega). 10 × 10^3^ cells were seeded in each well in a 96-well plate. Six concentrations of SEA and SEB (100, 50, 10, 5, 1, 0,1, 0.01 ng, 20 μl), with or without Cyt (20, 10, 5, 2,5, 1–0.1 μM, 10 μl) were used for cytotoxicity assays. To determine Cyt cytotoxicity before treatment with toxins, GDM-1, CESS, HL-60, QIMR-WIL, P31/FUJ and Kasumi-1 cell lines treated with six Cyt concentrations, separately. Then cells were treated with Cell Titer-Glo reagent, and the luminescence signal was detected using a microplate reader (FLUOstar Omega BMG Labtech, USA). Percent cell viability values were calculated for each cell line and in vitro Cyt IC50 values were calculated with R by using Six model (6 M) approach which was defined in our previous study (Supplementary Table S1) [24]. To determine toxins cytotoxicity cell lines were treated with seven SEA and SEB concentrations for 72 h, separately. For each toxin, proliferation rates were computed, and input files were generated for IC50 calculation with R. Since a decrease in cell proliferation was observed at 10 ng and there was no statistically significant difference between 5 ng and 10 ng, the experiments continued with 10 ng toxin. However, there was no toxicity at any concentration compared to the control. To determine the alterations of Cyt cytotoxicity after toxin treatment, cell lines were treated with 10 ng SEA and SEB for 72 h before Cyt treatment. After 72 h of treatment with toxins cell lines treated with Cyt concentrations. Percent cell viability values were calculated for each cell line and altered Cyt IC50 values were calculated with R by using 6 M [24].

#### 2.1.6 IC50 calculation

To calculate drug/toxin exposure variables like “IC50, EC50, activity area (AA), and Amax”, proliferations of cells was analyzed as a component of drug levels with/without toxin treatment using nonlinear regression, as described in The National Institutes of Health/NIH Chemical Genomics Center assay guidelines [25,26]. Although the nonlinear regression function used to delineate the inputs is commonly used in cytotoxicity measurements, we used six different models of this algorithm to calculate cytotoxicity values and choose the lowest standard error. With the 6 M approach IC50 values for GDM-1, CESS, HL-60, QIMR-WIL, Kasumi-1, and P31/FUJ cell lines treated with Cyt, SEA and SEB, were calculated separately by using own R script “SixModelIC50 V3.r” (https://github.com/muratisbilen/6-Model_IC50_CalculationV3.git) [24].

#### 2.1.7 Real-time Q PCR

CTSH, ATF5, HLA-DR4, AZU1, OAT and CD69 gene's expression was quantified using SYBR™ Green master mix (Biorad). PCR reactions were run under cycling conditions according to manufacturer's instructions. GAPDH was used as a reference gene in all reactions. qRT-PCR relative gene expression data was calculated using ddCT method. Primers used in this study are shown in Table 1.

**Table 1:** Primer sequences for selected genes.

Gene name	Forward Primer	Reverse Primer
ATF5	TGTCCTCGGATCACAGTCTCTT	TCAGAGAAGCCATCACCTGCC
AZU1	AGCTGCTTCCAAAGCCAGAAC	GTCGTAGCCATTCTCGCTCA
CD69	TGCCATCAGACAGCCATGTT	ACCCTGTAACGTTGAACCAGT
CTSH	TCATGGATGTCTAAGCACCGT	GCTCGATGAGGAAGTACCCG
HLA-DRB4	AGTACGCGCGCTACAACA	ATTCAGACCGTGCACTCCAT
OAT	GTTGCGCTTCATAGACGCC	GCACTGCAGACACAGGGTAT
GAPDH	GGAGCGAGATCCCTCCAAAAT	GGCTGTTGTCATACTTCTCATGG

### 2.2 In silico analyses

#### 2.2.1 In silico datasets and data normalization

To identify genes with differentially expressed, Cancer Genome Project (CGP- E-MTAB-783) gene expression data used from the ArrayExpress (https://www.ebi.ac.uk/arrayexpress/) and drug screening data was downloaded from the CGP database [27,28]. The raw data was Robust Multichip Average (RMA) normalized with the Affy package in the R/Bioconductor software.

#### 2.2.2 Spearman correlations

Spearman's correlation was used to test linear correlation between CGP IC50 data and in vitro IC50 data. Correlation coefficient (rho) and p values were calculated.

#### 2.2.3 Linear Models for Microarray (Limma) powers differential expression analysis

Differentially expressed genes were identified using Linear Models for Microarray (Limma) powers differential expression analysis to compare Cyt resistant and sensitive groups after treatment with SEA and SEB. Limma, R/Bioconductor has different tools for collecting data to address the issue of limited sample sizes [29]. To identify genes whose upregulation or downregulation may be involved in resistance to Cyt as well as increased migration/invasive properties, differentially expressed genes were compared between resistant group (HL-60, GDM-1, QIMR-WIL, and CESS) and sensitive (Kasumi-1 and P31/FUJ) group.

#### 2.2.4 Hierarchical clustering

In order to assess if the discovered genes could between resistant (HL-60, GDM-1, QIMR-WIL, and CESS) group and sensitive (Kasumi-1 and P31/FUJ) group, hierarchical clustering analysis was carried out. Prior to clustering, the mean and standard deviation of expression of genes were standardized to 0 and 1, respectively. Cluster 3.0 (http://bonsai.hgc.jp/∼mdehoon/software/cluster/software.htm) [30] was used to for hi-erarchical clustering using similarity metric parameter as Euclidean distance. Java Treeview (http://jtreeview.sourceforge.net/) [31] software was used for visualization of the clustering output.

#### 2.2.5 Network and pathway analyses

Pathway and network analyses were performed to assess the potential functional biological relevance of differentially expressed genes and their interrelationships. The GeneMANIA Cytoscape 3.9.1 software was used to show the network based on co-expression and genetic relationships between sensitive and resistant groups, and genes involved in related pathways were found in Cytoscape [32]. As input, 15 genes with differential expression were utilized. The datasets were examined and visualized to determine whether they included functionally connected genes and to determine important roles for certain gene groups in the network. Each gene is given a score by the program, and the size of the nodes has a direct correlation with the score. Higher scores represent genes with a greater potential for co-expression and a higher probability that they are functionally connected to one another. Purple lines represent co-expression and green lines represent genetic interaction between each gene. By using the Database for Annotation, Visualization and Integrated Discovery (DAVID) functional annotation tools, the pathways related to these genes were determined [33].

#### 2.2.6 Gene set enrichment (GSEA)

Gene set enrichment analysis (GSEA) was performed to evaluate biological processes that could be differentially active between resistant (HL-60, GDM-1, QIMR-WIL, and CESS) group and sensitive (Kasumi-1 and P31/FUJ) group based on the whole transcriptome. It was performed in accordance with the GSEA guideline process (http://software.broadinstitute.org/gsea/docGSEAUserGuideFrame.html) [34]. Gene sets having the same Gene ontology (GO) word annotations were found in the “C5 all Gene ontology v6.1 database,” which was utilized for the analysis. For gene set sizes, we selected the GSEA default filtering parameter, which contains genesets with sizes ranging from 15 to 500. GSEA is used to calculate the enrichment score (ES), normalized enrichment score (NES), nominal P value (NOM P value), false discovery rate q value (FDR q), and familywise error rate P value (FWER). We selected the enriched gene sets with FDR q values less than 0.25.

### 2.3 Statistical analysis

All statistical analyses were performed with GraphPad Prism software (San Diego, CA, USA). T-test (two tailed) was used to determine differences between toxin treated and nontreated cells, and values in the figures correspond to mean ± SD. A value of p and q < 0.05 was statistically significant.

## 3 Results

### 3.1 Effects of SEA and SEB on cell proliferation

**Fig. 1:**
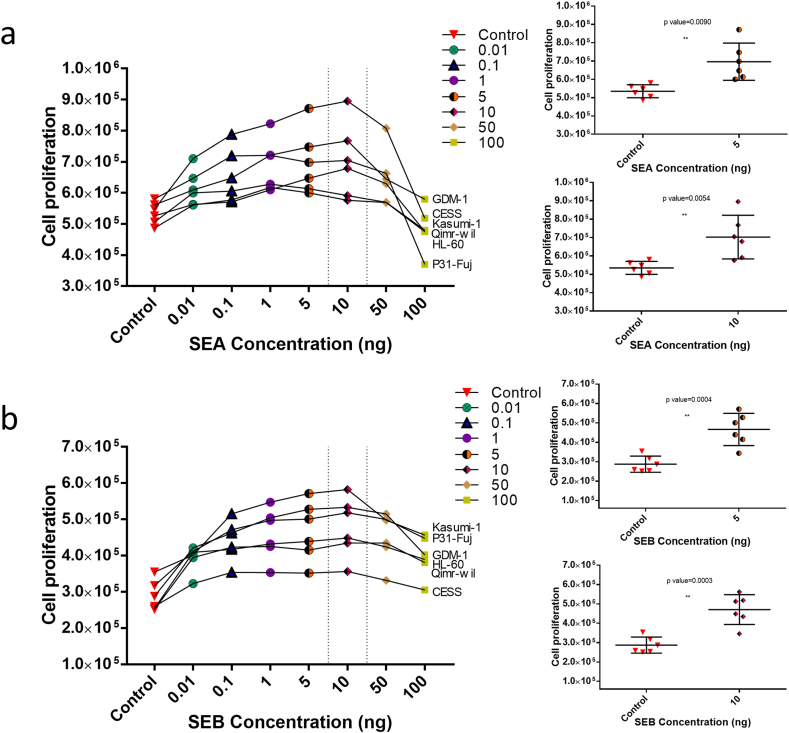
(a) Cell proliferation curves of GDM-1, CESS, HL-60, QIMR-WIL, P31/FUJ and Kasumi-1 cell lines after treated with six different concentration of SEA and (b) SEB for 72 h. Each experiment was performed with a total of three replicates. A statistically significant increase in cell proliferation is observed with both 5 ng toxin and 10 ng toxin compared to the control. For 5 ng, p: 0.0090 and 0.0004 (SEA and SEB respectively). And, for 10 ng, p value: 0.0054 and 0.0003 (SEA and SEB respectively).

An increase in growth percentages of all cell lines was observed after treatment with all six different concentrations of SEA and SEB (100, 50, 10, 5, 1, 0.1, 0.01 ng, respectively) compared to control (untreated). The highest cell proliferation of AML cell lines was seen after treatment with 5 ng and 10 ng of SEA (Fig. 1a) and SEB (Fig. 1b), respectively. Since a decrease in cell proliferation was observed at 10 ng and there was no statistically significant difference between 5 ng and 10 ng, the experiments continued with 10 ng toxin.

### 3.2 Collective AML cell migration and invasion in the presence of SEA and SEB

**Fig. 2:**
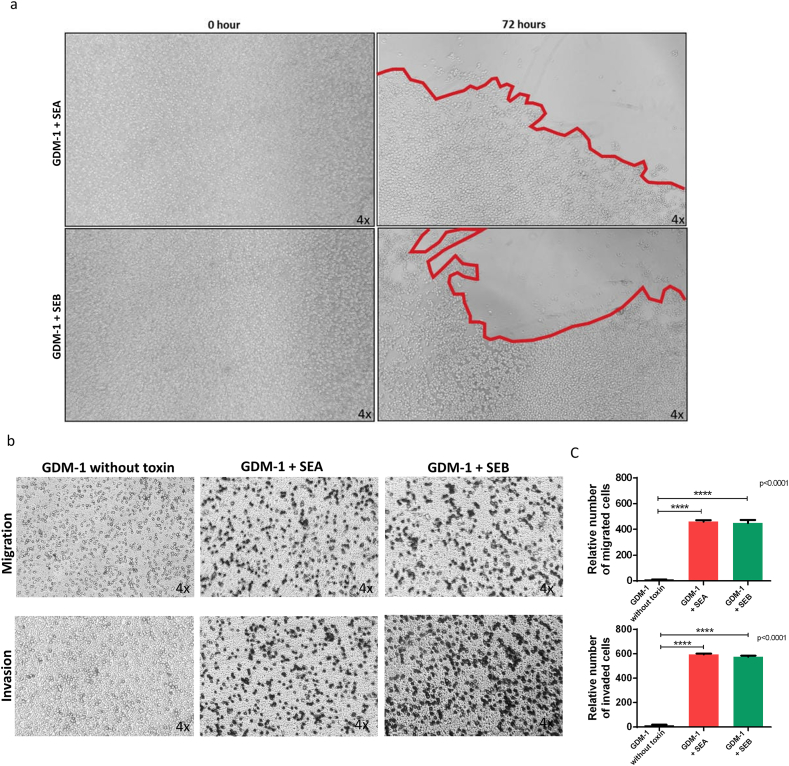
Cell migration and invasion assays of the GDM-1 cell line in the presence of SEA and SEB. (a) Figure shows the GDM-1 cell line treated with SEA and SEB at 0 and 72 h. Cells were conspicuously clustered at the edge of the well bordered by red lines at 72 h and no clustering at 0 h. (b) GDM-1 cells trying to migrate or invade after treatment with SEA and SEB were fixed in the filter pores. (c) Cells with a tendency to migrate or invade were counted and plotted against control. Migration and invasion experiments were performed with a total of three replicates and demonstrate that the GDM-1 cell line tends to migrate and invade approximately 50-fold compared to the control (n = 1) in a *t*-test, with p < 0.0001.

After 72 h of treatment with 10 ng of SEA and SEB, respectively, a tendency to migrate to the well edges was observed in all AML cell lines. As an example, Fig. 2a shows the migration of the GDM-1 cell line to well corners and formed edge margins under the microscope (4×).

At the end of the incubation period (72 h) of the migration experiments, all cells that migrated and adhered to the membrane were stained and counted with a hemocytometer. As an example, Fig. 2b shows stained GDM-1 cells migrating to the other side of the membrane after treatment with SEA and SEB, respectively.

More than fifty percent of AML cells in all six cell lines treated with enterotoxins tended to migrate, while only about one percent of untreated cells (control) were found to migrate. Therefore, the presence of SEA and SEB toxins significantly increased the migration and invasion capacities of AML cells by almost 50-fold. As an example, Fig. 2c shows relative migrated GDM-1 cells after treatment with SEA and SEB respectively compared to nontreated (control) cells.

Migration and invasion cell count results of all other AML cell lines are given in the supplementary figure (Supplementary Fig. S1).

### 3.3 Effects of SEA and SEB on Cyt cytotoxicity

**Fig. 3:**
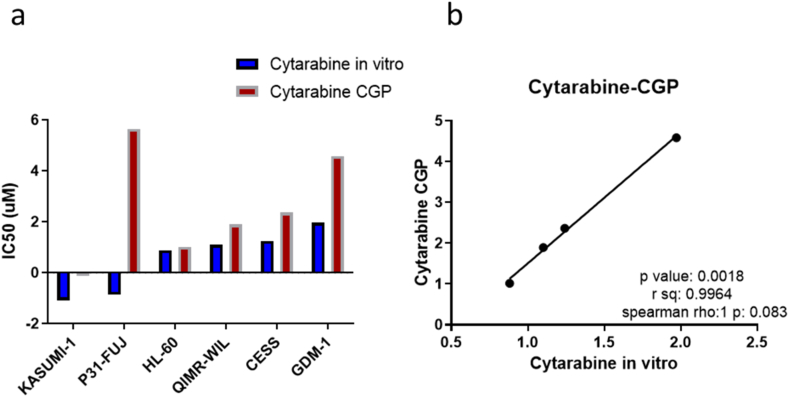
(a) CGP versus in vitro Cyt IC50 values for each AML cell line. (b) Spearman correlation between CGP Cyt IC50 drug data and in vitro Cyt IC50 data ranked from smallest to largest (log *(IC*_*50*_*) umol)*, and the Cyt correlation (Kasumi-1 and P31/FUJ excluded) (linear reg. p: 0.0018, r-sq.: 0.9964, rho:1, p: 0.083).

To test the effects of SEA and SEB on Cyt cytotoxicity, Cyt IC50 values were first obtained for all six AML cell lines (GDM-1, CESS, HL-60, QIMR-WIL, P31/FUJ and Kasumi-1) in the absence of toxins. Except for P31/FUJ values (Fig. 3a), it was determined that in vitro IC50's and in silico CGP IC50's correlated perfectly. This correlation becomes even more clear after excluding the marginal IC50 value of Kasumi-1 cell line (linear reg. p: 0.0018, r-sq.: 0.9964, rho:1, p:0.083) (Fig. 3b). P31/FUJ Cyt cytotoxicity experiments were repeated 3 times with new cells to see if this was due to any experimental error. Since similar results were obtained at the end of all 3 experiments, current IC50 values were used for subsequent analysis and experiments.

**Fig. 4:**
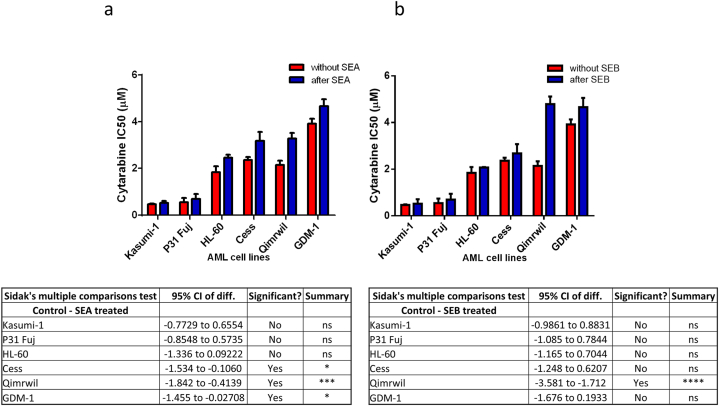
(a) In vitro Cyt IC50 values for each AML cell line before (no toxin) and after treatment with 10 ng SEA and (b) SEB for 72 h respectively. p value represents The p value represents the significance between the mean of six Cyt IC50 values of all AML cell lines before and after toxin treatment (*t*-test p value: 0.017 and 0.0313 for SEA and SEB).

Cyt IC50 values for each AML cell line were also calculated after treatment with 10 ng of SEA (Fig. 4a) and SEB (Fig. 4b) for 72 h, respectively. The result shows increased resistance to Cyt in all AML cell lines. However, these increases were not the same for all cell lines. Some cell lines (HL-60, CESS, GDM-1 and QIMR-WIL) became significantly more resistant. Using a two-way ANOVA, the IC50 values of SEA and SEB-treated cell lines were compared to those of the respective control groups. According to the results of Sidak's multiple comparison test, there is a significant increase in CESS, QIMR-WIL, and GDM-1 IC50s after treatment with SEA, but only QIMR-WIL after treatment with SEB.

According to the in vitro cytotoxicity profiles of six AML cell lines, we divided the cells into two groups; the resistant group (GDM-1, CESS, HL-60, QIMR-WIL) with an IC50 value higher than 2 μM and the sensitive group (P31/FUJ and Kasumi-1) with an IC50 value lower than 2 μM. A significant difference was determined when the IC50 values of these two groups were compared using *t*-test (two tailed) (ttest p < 0.01) (Supplementary Table S2).

### 3.4 Discovery of differentially expressed genes between resistant and sensitive groups

Limma analysis was performed to identify differentially expressed genes in these two groups. These differentially expressed genes are associated with higher resistance to Cyt after treatment with SEA and SEB in the resistant group (HL-60, GDM-1, CESS, QIMR-WIL).

**Fig. 5:**
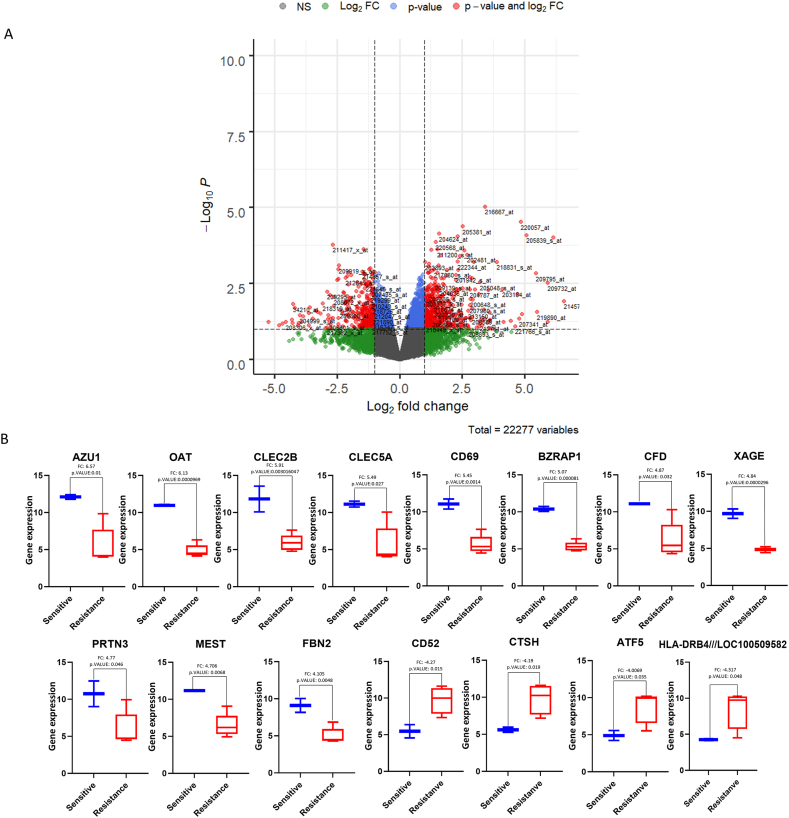
Differentially expressed genes in between resistant (HL-60, GDM-1, QIMR-WIL, and CESS) group and sensitive (Kasumi-1 and P31/FUJ) group. (a) Limma was used to compare resistant (n = 4) and sensitive (n = 2) groups of AML cell lines. 15 genes were differentially expressed (adj p-value <0.01, Log Fold change >4) between the two groups. (b) While 11 of these genes were down-regulated as shown in blue square boxes, four of these genes were found to be up-regulated in the resistant group as shown in red square boxes. NS: not significant, FC: fold change.

In order to determine the most significant differentially expressed genes (DEGs) with the highest fold change value between two groups, as a cut-off value, genes with a p value less than 0.05 and abs fold change value greater than 4 were selected. As a result, it was determined that there were 15 genes that could be related to higher resistance to Cyt (p-value <0.05, abs fold change >4) (Fig. 5a). While 11 of these genes were downregulated, 4 of these genes were upregulated in the resistant group (Fig. 5b).

**Fig. 6:**
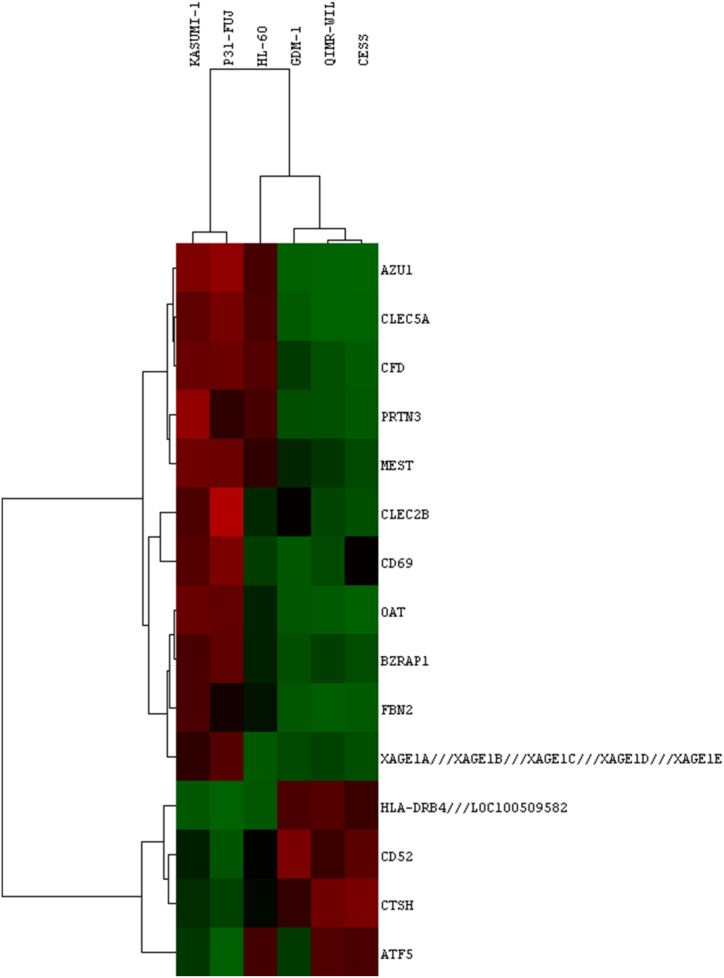
Hierarchical clustering of HL-60, GDM-1, QIMR-WIL, CESS, Kasumi-1 and P31/FUJ cell lines according to sensitivity profiles for Cyt with 15 genes. The analysis identifies subgroups for six AML cell lines that are sensitive (11 genes, green), and resistant (four genes, red). In two subgroups, sensitivity to Cyt is substantially correlated. Red represents high expression, whereas green represents low expression.

Additionally, hierarchical cluster analysis shows that these genes can distinctly differentiate the sensitive and resistant groups of AML cell lines (Fig. 6).

### 3.5 Biological features of differentially expressed genes with cellular functions

**Fig. 7:**
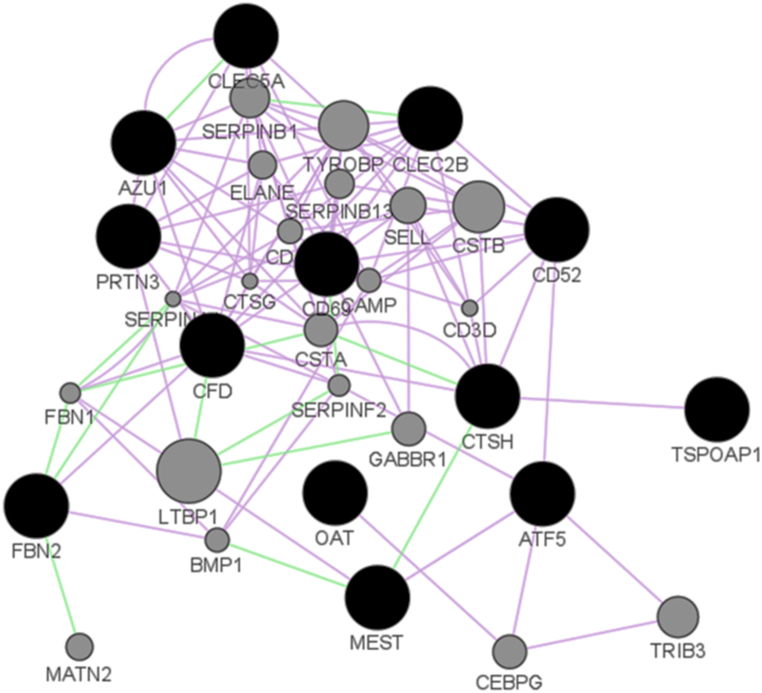
Functional network connectivity of 15 genes. Functional association of differentially expressed 13 out of 15 genes between resistant (HL-60, GDM-1, QIMR-WIL, and CESS) group and sensitive (Kasumi-1 and P31/FUJ) group. There is a strong network of co-expression and genetic interaction between these genes. Purple lines represent co-expression and green lines represent genetic interaction between each gene. Gray dots are other genes associated with these 13 genes identified by DAVID. Dot size correlates with close network.

An evaluation of the potential functional biological relevance of differentially expressed genes and their interrelationships revealed that 7 of these genes were involved in 3 immune related pathways (Table 2). The pathways in which these genes are located are shown in the table (Table 3). Likewise, figure shows that there is a strong network of co-expression and genetic interaction between all these 15 genes (Fig. 7).

**Table 2:** Pathway analysis of differentially expressed genes. Nearly half of the genes with differential expression between the two groups are involved in immune-related pathways.

Category	Term	Count	%	*P*-Value
REACTOME PATHWAY	Neutrophil degranulation	5 genes CLEC5A, AZU1, CTSH, CFD, PRTN3	41.7	0.0004
REACTOME PATHWAY	Innate Immune System	5 genes CLEC5A, AZU1, CTSH, CFD, PRTN3	41.741.7	0.0076
REACTOME PATHWAY	Immune System	7 genes CLEC5A, CLEC2B, AZU1, CTSH, CFD, HLA-DRB4, PRTN3	50.0	0.014

**Table 3:** The pathways in which 7 genes are located.

Gene Symbol	Gene Name	REACTOME_PATHWAY
**CLEC5A**	C type lectin domain containing 5A	Innate Immune System, Immune System, DAP12 interactions, Neutrophil degranulation,
**CLEC2B**	Ctypelectindomainfamily2 member	Adaptive Immune System, Immune System, Immunoregulatory interactions between a Lymphoid and a non-Lymphoid cell,
**AZU1**	Azurocidin 1	Innate Immune System, Immune System, Neutrophil degranulation
**CTSH**	Cathepain H	Adaptive Immune System, Innate Immune System, Immune System, MHC class I antigen presentation, Metabolism of proteins, Surfactant metabolism, Neutrophil degranulation,
**CFD**	Complement factor D	Hemostasis, Platelet degranulation, Complement cascade,I Initial triggering of complement, Innate Immune System, Immune System, Aternativecomplementactivation, Neutrephil degranulatian, Platelet activation, signaling and aggregation, Response to elevated plateletcytosolic Ca^2+^.
**HLA-DRB4**	HLA Class II Histocompatibility Antigen, DR Beta 4 Chain	Cytokine Signaling in Immune system, Adaptive Immune System, Immune System, TCR signaling, Downstream TCR signaling, Phosphorylation of CD3 and TCR zeta chains, Translocation of ZAP-70 to Immunological synapse, Generation of second messenger molecules, MHC class II antigen presentation, Costimulation by the CD28 family, PD-1 signaling, Interferon gamma signaling, Interferon Signaling,
**PRTN3**	Proteinage 3	Hemostasis, Cytokine Signaling in Immune system, Common Pathway of Fibrin Clot Formation, Formation of Fibrin Clot (Clotting Cascade), Innate Immune System, Immune System, Signaling by Interleukins, Other interleukin signaling, Neutrophil degranulation, Antimicrobial pepuces,

Geneset enrichment analysis show that 64 gene sets were significantly enriched in sensitive cells while 368 gene sets were significantly enriched in resistant group (FDR <25%). The first 20 gene sets enriched in sensitive and resistant groups respectively are presented in Supplementary Tables S3 and S4. GSEA results showed that most genesets significantly enriched in the resistant group were associated with innate and adaptive immune systems. These results are in parallel with pathway analysis. Fig. 8 shows the enrichment plots of the two most significantly enriched gene sets; adaptive immune response and lymphocyte activation in the resistant group (HL-60, GDM-1, QIMR-WIL, and CESS) compared to the sensitive (Kasumi-1 and P31/FUJ) group.

**Fig. 8:**
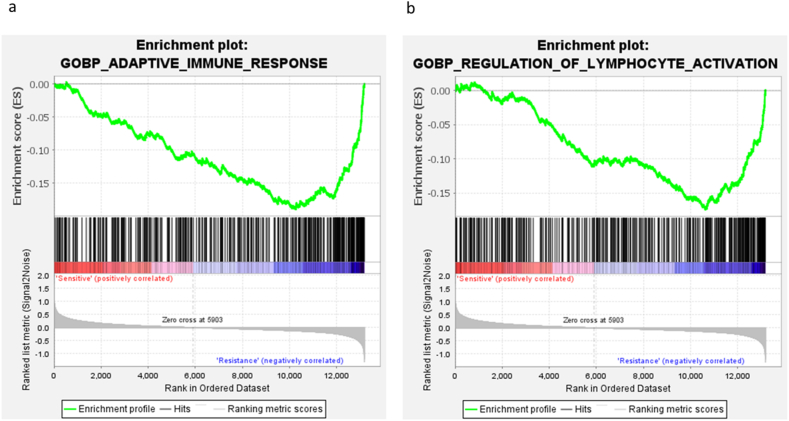
Comparative analysis of Gene set enrichment in drug-sensitive (Kasumi-1 and P31/FUJ) and resistant (HL-60, GDM-1, QIMR-WIL, and CESS) cell lines. (a) Enrichment analysis of adaptive immune response in drug-sensitive cell lines: panel delineates the enrichment plot for the adaptive immune response within drug-sensitive cell lines, namely Kasumi-1 and P31/FUJ. The y-axis represents the enrichment score, and the x-axis represents the rank in the ordered dataset. The annotation “sensitive” below the chart signifies a positive correlation with the adaptive immune response. (b) Enrichment analysis of regulation of lymphocyte activation in drug-resistant cell lines: Panel delineates the enrichment plot for the regulation of lymphocyte activation within drug-resistant cell lines, including HL-60, GDM-1, QIMR-WIL, and CESS. The y-axis delineates the enrichment score, and the x-axis represents the rank in the ordered dataset. The annotation “Resistance” below the chart indicates a negative correlation with the regulation of lymphocyte activation.

### 3.6 In vitro validation of differentially expressed genes between sensitive and resistant group

**Fig. 9:**
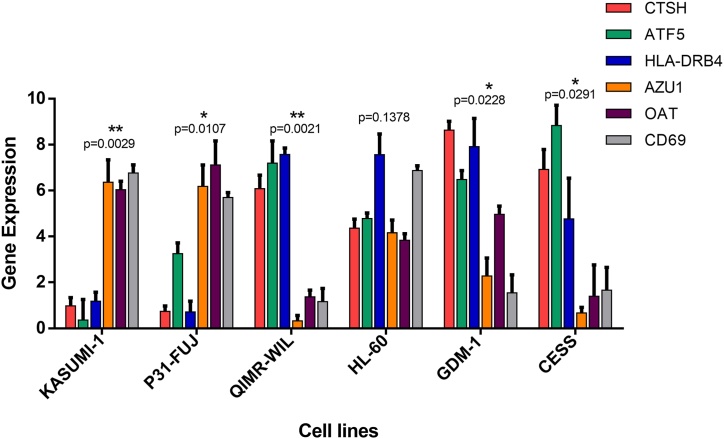
In vitro validation of differentially expressed CTSH, ATF5, HLA-DR4, AZU1, OAT and CD69 genes. We choose three upregulated CTSH, ATF5, HLA-DR4, and three downregulated AZU1, OAT and CD69 genes in resistant group for in vitro validation. CTSH, ATF5, HLA-DR4 genes were upregulated and AZU1, OAT and CD69 genes were downregulated in the resistant group by qRT PCR results (r > 0.8 p < 0.05).

We next validate the top six differentially expressed genes (three upregulated CTSH, ATF5, HLA-DR4, and three downregulated AZU1, OAT and CD69 in the resistant group) discovered within in silico analysis. Quantitative RT-PCR was used to evaluate the gene expression levels of six differentially expressed genes in resistant (HL-60, GDM-1, QIMR-WIL, and CESS) and sensitive (Kasumi-1 and P31/FUJ) cell lines. In vitro and in silico gene expression data were highly concordant, according to correlation analysis (Fig. 9). It was determined that CTSH, ATF5, HLA-DR4 genes were upregulated and AZU1, OAT and CD69 genes were downregulated in the resistant group by qRT PCR results (r > 0.8 p < 0.05).

## 4 Discussion

Staphylococcal enterotoxins are among the well known toxins for many years with their potent properties in cytokine release and fever formation [35]. Staphylococcal enterotoxin A and enterotoxin B are two types of toxins produced by the bacteria *S. aureus*, and both have been shown to have potential effects on cancer [36]. Both SEA and SEB, bind to MHC class II molecules, but it is well known that they have different structures and mechanisms of action, which may lead to different effects on cancer cells [14,37]. While both toxins have been reported to stimulate the growth and proliferation of certain types of cancer cells [38,39], some studies suggest that they may have anti-cancer effects against some types of cancers as well [40].

After elucidating the pathophysiological effects on the immune system and microenvironment, its effects on some cancers have also been the subject of research. Fooladi et al. published a systemic review on the potential of Staphylococcal enterotoxins to be candidates for cancer immunotherapy [41]. Staphylococcal enterotoxins are super antigens that can stimulate T cells at a significantly higher rate than other antigens (25 vs. 1/10000 of T cells). Therefore, they qualify as a suitable candidate for cancer immunotherapy. But for some types of cancer, the situation seems to be reversed. Primary cutaneous T cell lymphoma (PCTCL) is the prime example with very high (% 40) prevalence of *S. aureus* [42]. In addition to these data indicating that *S. aureus* may play a significant role in lymphoma pathogenesis, its eradication has also been shown to improve the disease [43]. This situation suggests that it increases in vivo immune dysregulation and cancer survival, has been partially proven by in vitro studies. Willerslev Olsen et al. showed that SEA activates oncogenic pathways in the cell line of cutaneous T-cell lymphoma and Sezary syndrome patients through in vitro stimulation of STAT3 and IL-17 [44]. Supportingly, Krejsgaard et al. showed that staphylococcal enterotoxins cause immune dysregulation because of STAT3 and IL-10 activation in the malignant clone, through crosstalk between benign and malignant T cells [45]. When these data are taken together, it can be assumed that activated gene pathways are the factor determining whether enterotoxin stimulation is for or against oncogenesis. In our study, we did not find any toxic effects of SEA and SEB on leukemia cell lines. Further studies are needed to explain why different tumor types develop different responses to *S. aureus* enterotoxins.

The weakened immune system of cancer patients increases their risk of infection [46]. This risk increases much further in bone marrow, where immune cells originate. The health of patients with blood malignancies such as leukemia and lymphoma are endangered by this situation [47]. It is well known that gram-positive bacteria, and particularly *S. aureus*, are responsible for a major percentage of emerging infections [48]. It is known that *S. aureus* produces over ten exotoxins, including enterotoxins [49,50]. After bacteria release these toxins into the environment, they can produce local or systemic damage by reacting with the blood where these toxins interact with leukemia cells [51]. Blood levels of Staphylococcal enterotoxin A (SEA) may vary based on the severity of the infection and the individual's immune system [48]. Although there is no precise report on the blood levels of these two enterotoxins, our findings indicate that both toxins can affect leukemic cells even in very low amounts (nm levels).

In this study, we showed how SEA and SEB, two important enterotoxins produced by *S. aureus*, can change proliferation, migration and invasion features of leukemia cell lines. After treatment with SEA and SEB, all AML cell lines showed an increase in proliferation, migration, and invasion, as well as Cyt resistance. However, we discovered that not all cells develop the same level of cytarabine resistance, and we identified the genes and pathways responsible. Interestingly, we identified a dysregulation in the expression of immune related genes in almost all cells that exhibited significant resistance after exposure to the toxin. In addition, pathway analysis revealed that differentially expressed genes are involved in crucial immunological pathways and as expected, are related with the progression of malignancy in various tumor types.

Cathepsin H (CTSH) is a member of the cathepsins superfamily as a lysosomal proteinase or endopeptidase with a distinct function. It belongs to a wide variety of protease enzyme subtypes and plays distinct roles such as angiogenesis, invasion in different tumorigenic processes. The role of cathepsins to malignant tumors invasion is well known [52].

The activating transcription factor 5 (ATF5) belongs to the family of cyclic adenosine monophosphate (cAMP) response element binding proteins. ATF5 has a crucial function in the control of cellular stress, as well as in cell differentiation, survival, and centrosome maintenance and development [53].

HLA-DRB5 is a paralogue of the HLA class II beta chain. This class II molecule is a heterodimer composed of a membrane anchored alpha and beta chain. By delivering peptides produced from extracellular proteins, it performs a key role in the immune system. Since the impact of MHC on murine leukemia was demonstrated in 1964, HLA relation has been considered a possible hereditary risk factor [54,55].

In their capacity as regulators of the innate immunity, granulocytes participate in the first phases of the immune response to malignancies. They are cytotoxic operators that work by releasing molecules such inflammatory cytokines, reactive oxygen species, cathepsin G, and azurocidin [56]. They may also contribute to the advancement of cancer by inducing angiogenesis and metastasis [57]. This gene produces the mitochondrial enzyme ornithine aminotransferase, a crucial enzyme in the route that transforms arginine and ornithine into glutamate and GABA, the primary excitatory and inhibitory neurotransmitters, respectively [58]. There are also indications that GABAergic signaling and its control on proliferation are not exclusive to the nervous system but are prevalent in organs harboring adult stem cells in the periphery. GABA has emerged as a peripheral tumor signaling molecule that regulates the growth of tumor cells and maybe cancer stem cells [59].

CD69 encodes a type II transmembrane receptor of the calcium dependent lectin class. Activation of T cells induces the expression of the encoded protein, which may play a role in proliferation. Multiple hemopoietic cells express CD69, which serves as an early activation marker in chronic lymphocytic leukemia [60]. Similarly, to the differentially expressed gene findings, the GSEA results demonstrate a considerable enrichment of immune related gene sets in the resistant group. A remarkable outcome of this study is the enrichment of gene sets related with antigen presentation. Dysregulation in the expression of determined genes indicates the loss of function of important immune cells in AML cases, as well as the inability to accurately and effectively express important immunological functions such as antigen presentation. However, as a result of our extensive research, we realized that these genes are not direc tly and specifically responsible for developing resistance to Cyt. At that time, we questioned the mechanism by which this resistance could develop.

The reaction of leukemic malignant cells to stress caused by the presence of enterotoxin in the environment may be the key to Cyt resistance. The cellular reaction to stress may either activate cell death pathways or sustain cell viability through an adaptive response. A cell, tissue, or organism that has been previously exposed to nonlethal stresses such as changes in temperature, oxygen and redox status, extracellular signals, and biochemical treatments such as chemotherapeutic drugs is more resistant to stress. During the adaptation process, cells undergo extensive metabolic and physiological changes to prevent cell damage and preserve homeostasis. This is accomplished by the coordinated action of many molecular signals, including autophagy, Endoplasmic reticulum (ER) stress signaling, and aging. These adaptive responses are crucial for carcinogenesis, tumor survival, and progression. ER stress begins a crucial survival and adaptability process. Almost all the genes that we validated in vitro which are significantly different between resistant and sensitive groups are genes associated with ER stress signaling. For example, Cai et al. showed that ER stress induced programmed cell death is antagonistically controlled by Catepsin B [61]. Targeting cathepsin C generates autophagic dysregulation that drives ER stress mediated cellular cytotoxicity [62].

While specific data on the direct effects of SEA and SEB treatment alleviating or exacerbating ER stress in the context of AML cell lines is not available, existing research has demonstrated that the presence of bacterial products can indeed trigger ER stress responses that contribute to the survival of cancer cells [63,64]. These studies suggest that ER stress may be a key factor in shaping cancer cell responses to bacterial toxins. In our study, although we did not directly investigate the modulation of ER stress by SEA and SEB, it is plausible that the observed alterations in proliferation, migration, and invasion could be influenced by the complex interplay between these toxins and ER stress signaling pathways. The connection between bacterial toxins, ER stress, and cancer cell behavior presents an intriguing avenue for further investigation. Future studies elucidating the precise interactions between SEA, SEB, and ER stress, and their contributions to AML cell behavior, could provide valuable insights into the underlying mechanisms. To comprehensively address the potential role of ER stress in the context of SEA and SEB treatment, additional experiments specifically probing ER stress markers and pathways would be necessary. Such investigations would further enhance our understanding of the interplay between bacterial toxins and cellular stress responses in AML cells.

Proteasome inhibition and ER stress upregulate ATF5 through PERK-mediated eIF2 phosphorylation. This increase in ATF5 may activate HSP27, an important molecular chaperone for survival and proper protein folding, in response to abnormal protein folding in the cytosol and ER [65]. Ulianich et al. demonstrated that ER stress reduces MHC-I expression on the surface of thyroid cells, which increases their vulnerability to NK-mediated lysis [66].

As mentioned in parallel to these genes we found immune related gen sets and pathways in our obtained results regard to increase in proliferation, invasion and migration of toxin treated AML cell lines. When ER proteostasis is disrupted by ER stress agents, a defensive response known as the unfolded protein response is triggered (UPR). Inducing transcriptional and translational processes to restore ER homeostasis is essential to the UPR's mission to ensure the survival of damaged cells. Many diseases, including cancer, inflammation, and degeneration, have been linked to abnormalities in the UPR signaling pathway. There is mounting evidence that ER stress plays a crucial role in controlling the outcome and the intensity of the immunological response. MHC I peptide presentation is controlled by protein synthesis and degradation, both of which are regulated by the unfolded protein response (UPR) [67,68]. Our study primarily utilized in vitro experiments with AML cell lines, and while we acknowledge the limitation of not conducting in vivo experiments to validate the effects of SEA and SEB, the inclusion of in vivo studies would provide a more comprehensive understanding of their impact on AML behavior in a complex physiological context. In vivo experiments are crucial for assessing the clinical relevance and potential translational applications of our findings.

## 5 Conclusions

*S. aureus* is the main cause of gram-positive bacterial infections in AML patients. Our results suggest that AML cell lines choose to adapt and escape from the undesirable environment formed in the presence of staphylococcal enterotoxins A and B. Meanwhile cells become more resistant to chemotherapeutic agents such as cytarabine. As a result of our transcriptomic analyzes and limited PCR validation experiments, we observed that some immune related genes, gensets and pathways were dysregulated in cells that became more resistant. When we searched for genes that were significantly different in the more resistant group and which we confirmed with PCR, we found that these genes were associated with ER stress, although they were associated with the immune system. This suggests that cells adapt to the undesirable environment through ER stress signaling and become more resistant. Therefore, initiating rapid treatment of infection in AML patients and evaluating the immune genes and pathways are important, considering their possible role in transforming cancer cells into more aggressive forms and developing drug resistance in the presence of even low levels of microbial toxins.

## Author contribution statement

Seyhan Turk: Conceived and designed the experiments; Performed the experiments; Analyzed and interpreted the data; Contributed reagents, materials, analysis tools or data; Wrote the paper.

Hatice Yanpar; Ayriana Safari Baesmat: Performed the experiments; Wrote the paper.

Secil Demirkol Canlı: Analyzed and interpreted the data.

Olgu Erkin Cınar: Contributed reagents, materials, analysis tools or data.

Umit Yavuz Malkan: Conceived and designed the experiments; Analyzed and interpreted the data; Contributed reagents, materials, analysis tools or data.

Can Turk: Conceived and designed the experiments; Performed the experiments; Analyzed and interpreted the data; Wrote the paper.

Ibrahim Celalettin Haznedaroglu; Gulberk Ucar: Analyzed and interpreted the data.

## Data availability statement

Data will be made available on request.

## Funding

This research was funded by HACETTEPE UNIVERSITY SCIENTIFIC RESEARCH CENTER, TCP-2019-18096.

## Declaration of competing interest

The authors declare that they have no known competing financial interests or personal relationships that could have appeared to influence the work reported in this paper.

## Figures and Tables

**figs1:**