# Supplementary material for: Enterotoxins A and B produced by Staphylococcus aureus increase cell proliferation, invasion and cytarabine resistance in acute myeloid leukemia cell lines
Source: Heliyon. 2023 Sep 2;9(9):e19743. doi: 10.1016/j.heliyon.2023.e19743 (PMC10559070; doi:10.1016/j.heliyon.2023.e19743)
Supplement: Multimedia component 2 [file mmc2.docx]

| S | S | R | R | R | R |  |  |
| --- | --- | --- | --- | --- | --- | --- | --- |
| KASUMI-1 | P31-FUJ | HL-60 | QIMR-WIL | CESS | GDM-1 | T test p val |  |
| -1,09 | -0,87 | 0,88 | 1,1 | 1,24 | 1,97 | 0,0032108 | s vs r |

**Table S2.** Cytarabine in vitro IC50 values of sensitive and resistant group.
