# Supplementary material for: Enterotoxins A and B produced by Staphylococcus aureus increase cell proliferation, invasion and cytarabine resistance in acute myeloid leukemia cell lines
Source: Heliyon. 2023 Sep 2;9(9):e19743. doi: 10.1016/j.heliyon.2023.e19743 (PMC10559070; doi:10.1016/j.heliyon.2023.e19743)
Supplement: Multimedia component 3 [file mmc3.docx]

| **GS** | **SIZE** | **ES** | **NES** | **NOM p-value** | **FDR q-value** | **FWER p-value** | **RANK AT MAX** |
| --- | --- | --- | --- | --- | --- | --- | --- |
| GOCC_AZUROPHIL-GRANULE | 125 | 0.29 | 3.77 | 0 | 0 | 0 | 3795 |
| GOCC_AZUROPHIL-GRANULE-LUMEN | 75 | 0.32 | 3.13 | 0 | 0,003 | 0,006 | 4655 |
| GOCC-SECRETORY-GRANULE -MEMBRANE | 236 | 0.17 | 2.99 | 0 | 0,006 | 0,019 | 3632 |
| GOCC-VESICLE-LUMEN | 289 | 0.15 | 2.93 | 0 | 0,007 | 0,028 | 5797 |
| GOCC-CYTOSOLIC-RIBOSOME | 75 | 0.27 | 2.8 | 0 | 0,014 | 0,065 | 8390 |
| COBP-CYTOPLASMIC-TRANSLATION | 111 | 0.23 | 2.78 | 0 | 0,014 | 0,078 | 8390 |
| GOBP-PROTEIN-DEGLYCOSYLATION | 21 | 0.5 | 2.7 | 0 | 0,021 | 0,126 | 3500 |
| GOCC-SPECIFIC-GRANULES | 136 | 0.2 | 2.68 | 0 | 0,02 | 0,137 | 2582 |
| HP-PSYCHOMOTOR-RETARDATION | 75 | 0.25 | 2.57 | 0 | 0,044 | 0,317 | 4654 |
| GOCC-POLYSOMAL-RIBOSOME | 26 | 0.42 | 2.55 | 0 | 0,048 | 0,362 | 7692 |
| GOBP-PROTEIN-FOLDING | 152 | 0.18 | 2.52 | 0 | 0,053 | 0,421 | 6293 |
| GOMF-TUBULIN-BINDING | 262 | 0.13 | 2.5 | 0,002 | 0,056 | 0,477 | 5465 |
| GOCC-AZUROPHIL-GRANULE-MEMBRANE | 43 | 0.33 | 2.47 | 0,002 | 0,066 | 0,571 | 3693 |
| HP-ABNORMAL-URINE-METABOLITE-LEVEL | 415 | 0.11 | 2.47 | 0 | 0,062 | 0,573 | 3601 |
| GOBP-REGULATION-OF-DNA-REPLICATION | 101 | 0.21 | 2.4 | 0 | 0,094 | 0,751 | 5264 |
| GOCC-SPECIFIC-GRANULE-MEMBRANE | 74 | 0.24 | 2.4 | 0,004 | 0,089 | 0,756 | 2388 |
| HP-RESTING-TREMOR | 46 | 0.3 | 2.38 | 0 | 0,095 | 0,806 | 6422 |
| GOCC-VACUOLAR-LUMEN | 142 | 0.17 | 2.38 | 0 | 0,093 | 0,815 | 3671 |
| GOMF-GAMMA-TUBULIN-BINDING | 25 | 0.4 | 2.38 | 0 | 0,089 | 0,818 | 2629 |
| HP-SUBMUCOSA-CLEFT-OF-SOFT-AND-HARD-PALATE | 49 | 0.28 | 2.33 | 0,002 | 0,115 | 0,909 | 7295 |

**Table S3.** Gene sets enriched in sensitive cell lines.
