# Supplementary material for: Enterotoxins A and B produced by Staphylococcus aureus increase cell proliferation, invasion and cytarabine resistance in acute myeloid leukemia cell lines
Source: Heliyon. 2023 Sep 2;9(9):e19743. doi: 10.1016/j.heliyon.2023.e19743 (PMC10559070; doi:10.1016/j.heliyon.2023.e19743)
Supplement: Multimedia component 4 [file mmc4.docx]

| **GS** | **SIZE** | **ES** | **NES** | **NOM p-value** | **FDR q-value** | **FWER p-value** | **RANK AT MAX** |
| --- | --- | --- | --- | --- | --- | --- | --- |
| GOBP_ADAPTIVE_IMMUNE_RESPONSE | 375 | -0,19 | -4,17 | 0 | 0 | 0 | 2911 |
| GOBP_REGULATION_OF_CELL_ACTIVATION | 483 | -0,16 | -4 | 0 | 0 | 0 | 2491 |
| GOBP_REGULATION_OF_LYMPHOCYTE_ACTIVATION | 375 | -0,17 | -3,89 | 0 | 0 | 0 | 2546 |
| GOBP_IMMUNE_RESPONSE_REGULATING_SIGNALING_PATHWAY | 326 | -0,18 | -3,74 | 0 | 0 | 0 | 2483 |
| GOBP_POSITIVE_REGULATION_OF_CELL_ACTIVATION | 299 | -0,18 | -3,59 | 0 | 0 | 0 | 2474 |
| GOBP_T_CELL_ACTIVATION | 414 | -0,15 | -3,59 | 0 | 0 | 0 | 2596 |
| GOBP_I_KAPPAB_KINASE_NF_KAPPAB_SIGNALING | 219 | -0,21 | -3,56 | 0 | 0 | 0 | 3661 |
| GOBP_ANTIGEN_RECEPTOR_MEDIATED_SIGNALING_PATHWAY | 147 | -0,25 | -3,43 | 0 | 0 | 0 | 2174 |
| GOBP_POSITIVE_REGULATION_OF_LEUKOCYTE_CELL_CELL_ADHESION | 210 | -0,2 | -3,34 | 0 | 0 | 0 | 2706 |
| GOBP_LEUKOCYTE_CELL_CELL_ADHESION | 314 | -0,17 | -3,29 | 0 | 0 | 0 | 2482 |
| GOBP_IMMUNE_RESPONSE_REGULATING_CELL_SURFACE_RECEPTOR_SIGNALING_PATHWAY | 213 | -0,19 | -3,27 | 0 | 0 | 0 | 2483 |
| GOMF_TUMOR_NECROSIS_FACTOR_RECEPTOR_BINDING | 28 | -0,52 | -3,26 | 0 | 0 | 0 | 2609 |
| GOBP_POSITIVE_REGULATION_OF_CELL_CELL_ADHESION | 247 | -0,18 | -3,26 | 0 | 0 | 0 | 3418 |
| GOBP_B_CELL_RECEPTOR_SIGNALING_PATHWAY | 53 | -0,38 | -3,21 | 0 | 0 | 0 | 2174 |
| GOBP_REGULATION_OF_T_CELL_ACTIVATION | 280 | -0,17 | -3,19 | 0 | 0 | 0 | 2546 |
| GOBP_ADAPTIVE_IMMUNE_RESPONSE_BASED_ON_SOMATIC_RECOMBINATION_OF_IMMUNE_RECEPTORS_BUILT_FROM_IMMUNOGLOBULIN_SUPERFAMILY_DOMAINS | 254 | -0,17 | -3,12 | 0 | 0 | 0,001 | 2781 |
| GOBP_POSITIVE_REGULATION_OF_I_KAPPAB_KINASE_NF_KAPPAB_SIGNALING | 147 | -0,22 | -3,1 | 0 | 0 | 0,001 | 3120 |
| GOCC_PLASMA_MEMBRANE_PROTEIN_COMPLEX | 467 | -0,12 | -3,09 | 0 | 0 | 0,001 | 6619 |
| GOBP_POSITIVE_REGULATION_OF_IMMUNE_RESPONSE | 416 | -0,13 | -3,04 | 0 | 0 | 0,006 | 2489 |
| GOBP_LEUKOCYTE_MEDIATED_IMMUNITY | 329 | -0,14 | -3,02 | 0 | 0 | 0,006 | 2904 |

**Table S4.** Gene sets enriched in resistant cell lines.
